# Physical, emotional and sexual adolescent abuse victimisation in South Africa: prevalence, incidence, perpetrators and locations

**DOI:** 10.1136/jech-2015-205860

**Published:** 2016-03-09

**Authors:** Franziska Meinck, Lucie D Cluver, Mark E Boyes, Heidi Loening-Voysey

**Affiliations:** 1 Department of Social Policy & Intervention, Centre for Evidence-Based Intervention, University of Oxford, Oxford, UK; 2 Department of Psychiatry and Mental Health, University of Cape Town, Cape Town, South Africa; 3 Health Economics and HIV/AIDS Research Division, University of KwaZulu-Natal, Durban, South Africa; 4 Health Psychology and Behavioural Medicine Research Group, School of Psychology and Speech Pathology, Curtin University, Perth, Western Australia, Australia; 5 UNICEF Office of Research Innocenti, Florence, Italy

**Keywords:** VIOLENCE, ADOLESCENTS CG, CHILD HEALTH, LONGITUDINAL STUDIES, PUBLIC HEALTH

## Abstract

**Background:**

Physical, emotional and sexual abuse of children is a major problem in South Africa, with severe negative outcomes for survivors. To date, no known studies have used data directly obtained from community-based samples of children to investigate prevalence, incidence, locations and perpetrators of child abuse victimisation. This study aims to investigate prevalence and incidence, perpetrators, and locations of child abuse victimisation in South Africa using a multicommunity sample.

**Methods:**

3515 children aged 10–17 years (56.6% female) were interviewed from all households in randomly selected census enumeration areas in two South African provinces. Child self-report questionnaires were completed at baseline and at 1-year follow-up (96.7% retention).

**Results:**

Prevalence was 56.3% for lifetime physical abuse (18.2% past-year incidence), 35.5% for lifetime emotional abuse (12.1% incidence) and 9% for lifetime sexual abuse (5.3% incidence). 68.9% of children reported any type of lifetime victimisation and 27.1% reported lifetime multiple abuse victimisation. Main perpetrators of abuse were reported: for physical abuse, primary caregivers and teachers; for emotional abuse, primary caregivers and relatives; and for sexual abuse, girlfriend/boyfriends or other peers.

**Conclusions:**

This is the first study assessing current self-reported child abuse through a large, community-based sample in South Africa. Findings of high rates of physical, emotional and sexual abuse demonstrate the need for targeted and effective interventions to prevent incidence and re-victimisation.

## Introduction

Evidence shows that victims of child abuse in sub-Saharan Africa have consistently poorer physical and mental health outcomes than other children.^1^ They are at increased risk for HIV infection,^2^ exposure to transactional sex,^3^ risk for re-victimisation,^4^ bullying^5^ and higher levels of depression and suicidal ideation.^6^ Little is known, however, about prevalence rates, perpetrators and locations of child abuse victimisation in South Africa, particularly among adolescents.

Adolescence is an important decade in a child's development, marking the period of transition from childhood to adulthood.^7^ Adolescents are a particularly vulnerable group, experiencing a third of all new HIV infections worldwide,^8^ high levels of violence, lower school attendance and enrolment than primary schoolchildren, early marriage and higher levels^9^ of sexual abuse victimisation.^10^ Furthermore, adolescence is a time where the intergenerational transmission of poverty, violence victimisation and perpetration, gender inequalities and educational disadvantage manifest themselves.^9^

Evidence of the epidemiology of adolescent abuse in South Africa is limited and inconsistent. Studies differ in sampling, and definitions or measures for severity of abuse.^11^ Consequently, reported prevalence rates vary greatly, 6.7–32% for physical abuse,^12^ ^13^ 11.9–35.5% for emotional abuse^12^ ^14^ and 1.6–60% for sexual abuse.^15^ ^16^ No published data are available on multiple abuse victimisation. Furthermore, existing evidence focuses on particular subpopulations of children. Specifically, past studies sampled selectively by including only one gender or by sampling exclusively university or high school students.^17^ These studies may be unrepresentative, given the low participation in higher grades of secondary school and tertiary education.^18^ Moreover, children who have dropped out of school may be at particularly high risk of abuse.^12^ Other studies assessed only high-risk children, such as orphans and vulnerable children (OVC)^12^ ^13^ and small-scale clinical case studies that examine clinical reviews of social services cases or children referred for psychological treatment.^19^ While these studies provide valuable evidence on subpopulations, wider community studies can help to understand broader prevalence rates and patterns. There is valuable research on outcomes of abuse and risk factors, using retrospective studies from adult samples,^15^ but these may need to be interpreted with caution in terms of determining prevalence rates due to risks of recall bias, particularly with regard to childhood memories of abuse.^20^ Finally, all known studies are either cross-sectional or retrospective. Therefore, they cannot measure incidence of abuse.

To the best of the authors’ knowledge, this study provides the first descriptive examination of prevalence, frequency, incidence, location, perpetrators and severity of child abuse in a longitudinal, community-based sample in South Africa. The study will focus on physical, emotional and sexual abuse victimisation. Community violence and other traumatic experiences outside of the home were excluded due to the study's use of the WHO definition of child abuse ‘in the context of a relationship of responsibility, trust or power’.^21^ Childhood neglect was not included due to high poverty within the sample, which made distinction between neglectful behaviour and poverty-related inability to provide difficult.^22^

### Objectives

This study aims to assess physical, emotional, sexual and multiple child abuse victimisation within a large, random, community-based sample of adolescents in terms of: (1) prevalence and frequency; (2) incidence; (3) differences by gender, age and rural/urban location; and (4) perpetrators and locations of victimisation.

## Methods

### Participants

Recruitment took place in two urban and rural health districts in two provinces: Mpumalanga and the Western Cape. In each district, census enumeration areas were randomly selected. Within these areas, every resident child aged 10–17 years (n=3514) was included through door-to-door sampling of all households. In households with several children, one participant was selected randomly. Baseline data were collected between January 2010 and June 2011. A year later, 3401 (96.7%) participants were traced and reinterviewed.

### Procedures

Child participants completed confidential questionnaires which were translated into Xhosa, Swati, Tsonga, Sotho and Zulu and checked with back translation. Interviewers assisted participants in filling in the questionnaires, which took 60 min to complete. Children were interviewed in the language and locations of their choice, such as spare classrooms in schools or under a secluded tree, to ensure confidentiality. Interviewers received intensive training in working with vulnerable children and in administering standardised questionnaires. Vulnerable youth pre-piloted the survey to assess age-appropriateness and cultural-appropriateness. Data were checked for quality and missing data were <0.5%.

Ethical approval was granted by the Universities of Oxford, Cape Town and KwaZulu-Natal; the National Department of Social Development; and the Western Cape and Mpumalanga provincial Departments of Health and Education. Informed consent was sought from both children and their caregivers. Within sub-Saharan Africa, many children are not looked after by their biological parents. The term ‘caregivers’ is therefore used and refers to the person who provides primary parenting responsibilities. Caregivers can be biological parents, aunts and uncles, siblings, other relatives or foster carers. Information and consent sheets were read out loud and questions answered in the light of low literacy in the sampled population group. Participation was voluntary, and all participants received a certificate of completion and light refreshments.

Children at risk of significant harm, as well as those with past experiences of abuse or who requested help, were referred to local child protection services, counselling centres and HIV-testing services with follow-up support from the interviewers. These options were always discussed with the child, otherwise strict confidentiality was maintained. In total, 664 referrals were made.

### Measures

Child physical and emotional abuse victimisation were measured (at both baseline and follow-up assessments) using five items from the UNICEF Measures for National-level Monitoring of OVC.^23^ Participants were asked to state frequency of abuse in the past year (never, happened but not past year, at least once, monthly and weekly). The scale had been used in South Africa previously and showed good reliability of α=70.^12^ Seven additional items were designed and tested for follow-up data collection with the help of local social workers, NGO staff working with OVC, and adults and children from the local community (all items are listed in online supplementary material 1; for frequencies of responses to individual items, please see Meinck *et al*^24^). All follow-up items also measured the relationship of the perpetrator to the child and the location of the abuse. The overall reliability for this 14-item scale was α=0.74.

10.1136/jech-2015-205860.supp1 Supplementary data

*Child sexual abuse victimisation* at baseline was measured using two items designed by social workers in South Africa and one item from the National Survey of HIV and Risk Behaviour Amongst Young South Africans.^25^ All baseline sexual abuse items measured lifetime exposure with a no/yes response code. Sexual abuse victimisation at follow-up was measured in more detail using five items from the Juvenile Victimization Questionnaire.^26^ Items were modified to fit the cultural context with the help of experienced social workers and were then pre-piloted with children in South Africa (all items are listed in online supplementary material 2). Contact sexual abuse was defined as any unwanted touching or kissing, touching of private parts and/or forced sex. Exposure to sexual harassment and forced watching of pornographic material were also measured.

10.1136/jech-2015-205860.supp2 Supplementary data

For all abuse items administered at follow-up and for physical and emotional abuse at baseline, participants were asked to state frequency of abuse in the past year (never, happened but not past year, at least once, monthly and weekly). All follow-up items also measured the relationship of the perpetrator to the child and the location of the abuse.

To estimate the incidence of the three types of abuse, individual scales for physical, emotional and sexual abuse were coded into dichotomous variables. If the child did not report victimisation at baseline or at follow-up, or if the child mentioned victimisation at both baseline and follow-up, the response was coded as 0. If victimisation was mentioned at follow-up but not at baseline, this was coded as 1. To allow comparability across the two measurement points, exactly the same items were used at baseline and follow-up to determine incidence for physical and emotional abuse. Similarly, dichotomous variables were created for prevalence at baseline and follow-up in each abuse category (0: not abused; 1: abused). Prevalence was further divided to reflect lifetime abuse, past year and monthly or more frequent victimisation.

Perpetrators and locations of abuse victimisation were identified by participants for each abusive act they had experienced. The options were: caregiver, teacher, relative, neighbour or ‘other’. For ‘other’, further clarification on the perpetrator's relationship with the child was requested. Participants also had the option of indicating the location in which they had been victimised. The options were: home, community, school, home of neighbour and ‘other’. For ‘other’, further clarification of the location was requested.

*Multiple abuse victimisation* was defined as two or more categories of abuse, and measured as two or more concurrent abuse types (of sexual, physical and emotional abuse).

*Sociodemographic* information on gender, age, province and location (urban or rural) was measured using items modelled on the South African census.

### Analyses

Descriptive analyses were conducted using SPSS V.22. Estimates of incidence and prevalence of physical, emotional, sexual abuse and multiple abuse victimisation were assessed. Perpetrators and locations of perpetration were also examined. Prevalence rates were tested for demographic differences using Pearson's χ^2^-tests.

## Results

The sample included 3515 children at baseline (56.7% female, mean age 13.45 years) and 3401 (96.7% retention rate) at follow-up (54.5% female, 14.67 years). Refusal rates were 2.8% at baseline and <0.5% at follow-up. Approximately half of the participants lived in urban areas (50.6% at baseline, 48.6% at follow-up) and in Mpumalanga Province (47.3%).

### Prevalence and incidence

In total 68.9% of children reported at least one type of victimisation in their lifetime and 54.9% reported some type of victimisation in the past year. In total 32.3% of children reported at least one type of frequent monthly abuse victimisation (table 1).

**Table 1:** Prevalence rates of physical, emotional and sexual child abuse victimisation

	Never % (n)	Lifetime % (n)	In the past year %(n)	Monthly % (n)	Incidence % (n)
Physical abuse					
Baseline	59.9 (2036)	–	40.1 (1365)	18.2 (619)	–
Follow-up	43.7 (1485)	56.3 (1916)	37.9 (1289)	16.6 (564)	18.2 (641)
Emotional abuse					
Baseline	65.8 (2237)	–	34.2 (1160)	19.2 (654)	–
Follow-up	64.5 (2195)	35.5 (1206)	31.6 (1076)	20.7 (704)	12.1 (410)
Sexual harassment					
Baseline	–	–	–	–	–
Follow-up	85.7 (2899)	14.8 (502)	12.8 (437)	8.1 (276)	–
Forced exposure to pornography					
Baseline	–	–	–	–	–
Follow-up	97.6 (3319)	2.4 (82)	2 (69)	0.8 (26)	–
Contact sexual abuse					
Baseline	96.3 (3276)	3.7 (125)	–	–	–
Follow-up	89.8 (3054)	9 (306)	5.9 (201)	2.8 (94)	5.3 (181)
Rape					
Baseline	98.8 (3360)	1.2 (41)	–	–	–
Follow-up	96.7 (3290)	3.3 (111)	0.8 (28)	0.3 (10)	2.1 (70)
Any type of victimisation experienced					
Baseline	46.1 (1567)	53.9 (1834)	–	–	–
Follow-up	31.1 (1057)	68.9 (2344)	54.9 (1868)	32.3 (1100)	–
Multiple abuse victimisation					
Physical and emotional	72.9 (2479)	27.1 (922)	19.6 (667)	9.2 (312)	–
Physical and sexual	93.9 (3192)	6.1 (209)	3.6 (122)	0.8 (28)	–
Emotional and sexual	94.4 (3211)	5.6 (190)	3.6 (123)	1.6 (56)	–
Physical, emotional and sexual	95.4 (3246)	4.6 (155)	3.2 (109)	0.6 (22)	–

*Physical abuse:* At follow-up, 56.3% of children reported lifetime physical abuse, 37.9% reported past-year physical abuse and 16.6% reported frequent monthly physical abuse victimisation. Past-year incidence of physical abuse was 18.2%.

*Emotional abuse:* In total 35.5% of children reported lifetime emotional abuse, 31.6% reported past-year emotional abuse and 20.7% reported frequent monthly emotional abuse victimisation. Past-year incidence of emotional abuse was 12.1%.

*Sexual abuse:* At follow-up, 14.8% of children reported lifetime sexual harassment and 12.8% reported sexual harassment in the past year. Up to 2.4% reported lifetime forced exposure to pornography, and 2% reported forced exposure to pornography in the past year. Nine per cent of children reported lifetime contact sexual abuse, 5.9% reported past-year contact sexual abuse exposure, and 2.8% reported frequent monthly sexual abuse victimisation. Up to 3.3% of children reported lifetime rape, 0.8% reported past-year rape, and 0.3% reported frequent monthly rape victimisation. Past-year incidence of contact sexual abuse was 5.3%; past-year rape incidence was 2.1%. Since sexual harassment and exposure to pornography were not measured at baseline, incidence of either was not calculated.

*Multiple victimisation:* Up to 27.1% reported being victims of two or more types of abuse victimisation in their lifetime, with physical and emotional abuse most commonly co-occurring. Up to 19.6% reported frequent multiple victimisation.

### Children at higher risk for abuse

Demographic differences in abuse victimisation were also observed (table 2). Younger children were more likely to experience physical abuse (p<0.001), while older children were more likely to report emotional (p<0.05) and sexual abuse (p<0.001). Girls were more likely than boys to report emotional abuse (p<0.005), sexual harassment (p<0.001), contact sexual abuse (p<0.001) and rape (p<0.001). In addition, children in rural areas were more likely to report physical (p<0.005), emotional (p<0.05) and contact sexual (p<0.05) abuse victimisation than those in urban areas. However, the differences were not as distinct as for other demographic factors. There were no differences between genders for physical abuse and exposure to pornography. Likewise, no differences between urban and rural sites could be observed for sexual harassment, exposure to pornography and rape victimisation. Perpetrators and locations of abuse.

**Table 2:** Differences in abuse victimisation by gender, location and age

	Male (n=1475)	Female (n=1926)	Urban (n=1720)	Rural (n=1681)	Age <15 (n=2234)	Age 15+ (n=1167)
Physical abuse						
Past year	17% (577)	20.9% (712)	17.6% (600)	20.3% (689)**	20.3% (691)	17.6% (589)***
Lifetime	25.2% (856)	31.2% (1060)	27.8% (946)	28.5% (970)	29.1% (988)	27.3% (928)***
Emotional abuse						
Past year	12.6% (428)	19.1% (648)**	15% (509)	16.7% (567)*	14% (475)	17.7% (601)*
Lifetime	14.3% (458)	21.2% (721)**	16.9% (576)	18.5% (630)*	15.8% (538)	19.6% (668)*
Sexual harassment						
Past year	3.1% (106)	9.7% (331)***	6.1% (207)	6.8% (230)	3.6% (122)	9.3% (315)***
Lifetime	3.7% (127)	11% (375)***	7% (239)	7.7% (263)	4.3% (145)	10.5% (357)***
Pornography						
Past year	0.7% (25)	1.3% (44)	0.8 (27)%	1.2% (42)	0.7% (24)	1.3% (45)
Lifetime	0.9% (29)	1.6% (53)	1.1% (36)	1.4% (46)	0.9% (29)	1.6% (52)*
Contact sexual abuse						
Past year	1.8% (61)	4.1% (140)***	2.5% (84)	3.4% (117)*	1.8% (60)	4.1% (141)***
Lifetime	3% (101)	6% (205)***	4.1% (140)	4.9% (166)	2.6% (87)	6.4% (210)***
Rape						
Past year	0.1% (3)	0.7% (25)***	0.4% (14)	0.4% (14)	0.3% (9)	0.6% (19)
Lifetime	0.9% (29)	2.4% (82)***	1.8% (60)	1.5% (51)	0.8% (27)	2.5% (84)***

Pearson's χ^2^ test (two-tailed): p<0.001 ***, p<0.01**, p<0.05*.

Perpetrators of physical abuse victimisation were most commonly primary caregivers, followed by teachers and relatives. Perpetrators of emotional abuse were most commonly primary caregivers, followed by relatives and teachers. Perpetrators of sexual abuse (harassment, forcing participants to watch pornography, unwanted sexual touching or kissing, unwanted genital touching) were most commonly peers and intimate partners. Forced sex was mainly perpetrated by strangers, relatives and intimate partners. The most common locations for physical and emotional abuse were the home, followed by schools and communities. The locations for sexual harassment, being forced to watch pornography, unwanted sexual touching and unwanted genital touching were primarily the community and school. Forced sex was mostly reported to have happened in the home and at school (table 3).

**Table 3:** Perpetrators and locations of child abuse victimisation

Perpetrators	Percentage	Locations	Percentage
Physical abuse			
Caregiver	47.4	Home	58.6
Teacher	32.4	School	36.1
Relative	11.4	Community	4.7
Neighbour	1.7	Home of neighbour	0.2
Other	7.1	Other	0.1
Emotional abuse			
Caregiver	56.9	Home	76.2
Teacher	5.9	School	12.2
Relative	19.3	Community	9.8
Neighbour	6.4	Home of neighbour	1.3
Other	11.5	Other	0.5
Sexual abuse			
Caregiver	2.2	Home	11
Teacher	0.9	School	32.1
Relative	7.5	Community	52.6
Neighbour	13.2	Home of neighbour	1.2
Boyfriend/girlfriend	24.1	Other	3.1
Peers	30.9		
Stranger	14.9		
Other	6.3		
Rape			
Caregiver	1.9	Home	58.6
Teacher	0	School	36.1
Relative	23.1	Community	4.7
Neighbour	9.6	Home of neighbour	0.4
Boyfriend/girlfriend	23.1	Other	0.3
Peers	7.9		
Stranger	28.8		
Other	5.8		

## Discussion

This is the first large-scale community-based study examining the incidence and prevalence of current self-reported child abuse victimisation in South Africa. It adds valuable information to the existing literature regarding perpetrators, locations, incidence and prevalence rates of physical, emotional and sexual child abuse. In addition, no other published study in South Africa has investigated multiple abuse victimisation. This is important, since research in other countries has shown that large numbers of child abuse victims are subject to multiple types of abuse^27^ and have especially poor physical and mental health outcomes.^28^

### What are the prevalence and incidence rates of physical, emotional and sexual child abuse in South Africa?

Overall, participants reported a high prevalence of lifetime, past-year and frequent physical, emotional and contact sexual abuse victimisation. A high incidence of physical, emotional and contact sexual abuse was also reported. Up to one-third of participants reported lifetime multiple abuse victimisation. Girls and older children were found to be at particular risk for sexual and emotional abuse. Younger children were at higher risk for physical abuse victimisation.

Comparisons of child abuse victimisation are notoriously difficult due to measurement issues.^29^ However, the present study and official reports suggest very high rates of child abuse victimisation compared to those in high-income countries.^30^

### What sociodemographic factors put children at higher risk of abuse?

While younger children experience more physical abuse, older children were more likely to report emotional and sexual abuse. This finding seems broadly in line with the literature from other studies in Africa,^11^ such as the Violence Against Children studies,^31^ ^32^ as well as studies from high-income countries.^33–35^

Gender also matters in abuse victimisation. Girls reported more emotional and lifetime sexual abuse than boys. These findings correspond to global^36^ and African evidence.^31^ ^32^ For emotional abuse, only one study in South Africa has previously examined gender differences^13^ (most surveys have been single gender), and evidence from other countries is inconclusive.^33^ Interestingly, substantial differences in abuse victimisation between children in rural and urban areas could not be found, and previous findings were also inconclusive.^11^

### Who are the perpetrators, and what locations do they use?

Findings show differences between the perpetrators of physical, emotional and sexual abuse. Caregivers and teachers committed the majority of physical abuse. Since caregivers are usually the child's main disciplinarian, spend a large amount of time with the child and may consider physical discipline a behavioural measure,^37^ it is not surprising that they are the main perpetrators of physical and emotional abuse.^38^ Consistent with previous evidence, physical punishment by teachers remains common in schools across the country despite the abolition of corporal punishment in schools.^39^

The most common perpetrators of rape were strangers, peers and relatives, a finding which corresponds to findings from other studies in South Africa.^15^ ^17^ Contact sexual abuse and sexual harassment were carried out mostly by peers/friends or relatives, findings which are consistent with other South African studies.^39^ Contrary to other studies in South Africa, few children reported teachers as sexual abusers.^40^

### Limitations

This study was subject to a number of limitations. First, it took place in low-income black African communities, and thus the observed prevalence rates cannot be generalised across high-income areas and other ethnic groups within South Africa. However, 54% of black South Africans live under the poverty line,^41^ and the study benefited from in-sample variation such as the inclusion of five African language groups and the administering of questionnaires in rural and urban areas in two provinces. Second, data were collected using child self-report only. Evidence suggests that children routinely under-report their experiences of abuse.^42^ Studies that include parents have shown, however, that parents are even more likely to under-report abusive behaviours towards their children,^43^ and use of social services cases or substantiated court reports are unreliable in contexts where services are unable to reach the majority of abuse victims. Furthermore, current child self-report is preferable to the routinely used retrospective measures.^44^ In addition, measures were carefully piloted, included in a removable confidential section of the questionnaire, and interviews were conducted with particularly sympathetic interviewers. Third, no information on the perpetrators’ gender was collected. Future studies should investigate perpetrator gender in order to inform targeted interventions aimed specifically at sexual abuse perpetrators. Finally, the study focused on the prevalence and incidence of physical, emotional and sexual abuse victimisation only and did not measure neglect or any forms of community violence. Experience of more than one type of child abuse victimisation as defined in this study is therefore classified as multiple abuse victimisation rather than polyvictimisation. Results should be interpreted with this in mind.

### Implications

Current child abuse prevention efforts often focus on younger children, but our results suggest that adolescents are also vulnerable to abuse victimisation (with 32.3% of the adolescents in this sample experiencing at least one type of frequent abuse victimisation). This suggests that broadening child protection efforts is necessary to ensure that this age group is reached. The findings also demonstrate that child abuse appears to be prevalent across genders, locations and settings despite some differences between boys and girls and between younger and older adolescents. While the current child protection system in South Africa mainly focuses on responding to child abuse, policymakers and practitioners should consider investing in child abuse prevention programmes in the light of these findings. Home visiting, evidence-based parenting programmes and multicomponent interventions have been shown to be effective in other parts of the world.^45^ Given the wide number of contexts in which abuse occurs, these services should be integrated with education, family health services such as maternal health, early childhood development, immunisations and adolescent health services as suggested by a recent Child Maltreatment Readiness Assessment in South Africa.^46^

### Directions for future research

Research investigating risk and protective factors for child abuse victimisation is needed to inform intervention design and programming. Further research is required to examine social and familial predictors of abuse in order to identify modifiable pathways. Since existing family interventions focus primarily on parents with infants or young children, future research should consider the design and evaluation of child abuse prevention interventions for families with older children and adolescents. Furthermore, the role of gender (for both the child and the perpetrator) in sexual abuse victimisation needs to be investigated.

Future research should also investigate risk factors for multiple abuse victimisation in South Africa. Child abuse prevention efforts should consider the importance of co-occurrence of several types of abuse, since studies have shown that multiple childhood abuse victims are at a higher risk of developing trauma symptomology and are more likely to experience severe abuse.^47^

## Conclusion

The incidence and prevalence rates of physical, emotional and sexual abuse in South Africa are high in comparison to Western samples and similar to rates shown in other sub-Saharan African countries. Many children also report multiple abuse victimisation. Perpetrators of physical abuse are mainly caregivers and teachers; perpetrators of emotional abuse are mainly caregivers and relatives; and perpetrators of sexual abuse tend to be intimate partners and peers.

The findings of this study have implications for policy and intervention design in South Africa. They suggest the need for targeted child abuse prevention interventions and service provision. Such programmes should take into account the heterogeneity in victims, perpetrators and locations across the different types of abuse.

**What is already known on this subject**

- Physical, emotional and sexual child abuse victimisation has been shown to influence health in later life. Recent studies have found that the prevalence of child abuse victimisation is high across sub-Saharan Africa, but existing studies focus exclusively on school or university students, or on only one gender. They are cross-sectional and therefore cannot determine incidence.

**What this study adds**

- This longitudinal study examined physical, emotional, sexual and multiple abuse victimisation in a community-based sample of South African adolescents. It identified incidence and prevalence of abuse victimisation as well as perpetrators and locations. The results emphasise the great importance of targeted interventions for abuse response services and prevention.
