# Supplementary material for: Physical, emotional and sexual adolescent abuse victimisation in South Africa: prevalence, incidence, perpetrators and locations
Source: J Epidemiol Community Health. 2016 Mar 9;70(9):910–6. doi: 10.1136/jech-2015-205860 (PMC5013157; doi:10.1136/jech-2015-205860)
Supplement: Supplementary data [file jech-2015-205860supp1.pdf]

Supplement 1: Questionnaire items for Child physical and emotional abuse victimisation

**Source:**	**Questionnaire items:**
UNICEF Measures for National-level Monitoring of Orphans and Other Vulnerable Children	
	How often in the past year did an adult…
	1) use a stick, belt or other hard item to hit you?
	2) slap, punch or hit you so that it hurt?
	3) threaten to send you away or kick you out of the house?
	4) threaten to invoke ghosts or evil spirits or harmful people?
	5) call you dumb, lazy or other names?
	6) withhold a meal to punish you?*****
	7) single you out to do household chores all day instead of school or play?*****
Items design by local social workers, NGO staff, children from the local community.	
	8) make you stand or kneel in an uncomfortable position for a long period of time to punish you?*
	9) threaten to hurt you or give you bad grades?*
	10) insult members of your family that have passed away?*
	11) tell you t hey wished they did not have to look after you or make you feel you are a burden
	12) threaten to leave you and never come back?*
	13) make you feel unwelcome at home?*
	14) threaten to hurt or kill a person or an animal that you care about?*

*Administered at follow-up assessment only.
