# Supplementary material for: Physical, emotional and sexual adolescent abuse victimisation in South Africa: prevalence, incidence, perpetrators and locations
Source: J Epidemiol Community Health. 2016 Mar 9;70(9):910–6. doi: 10.1136/jech-2015-205860 (PMC5013157; doi:10.1136/jech-2015-205860)
Supplement: Supplementary data [file jech-2015-205860supp2.pdf]

Supplement 2: Questionnaire items for child sexual abuse victimisation

Question:

Baseline:

National Survey of HIV and Risk Behaviour:

"Have you ever had sex with someone when you didn't want to because they hurt you, or you were afraid that they were going to hurt you if you didn't?"

Local social workers:

Has anyone ever…

1) touched you in a way that made you feel uncomfortable?

2) made you do something with your private parts or their private parts that you did not want to?

Follow-up:

Juvenile Victimization Questionnaire

How often in the past year did someone...

1) tell you that you look sexy in a way that made you feel uncomfortable?

2) force you to watch sexual things or pictures with nude images?

3) touch or kiss you in a way that made you feel uncomfortable?

4) touch your private parts or asked you to touch their private parts even though you did not want this to happen?

5) force you to have sex with them in any way when you did not want to?
